# Should Cognitive Screening Tests Be Corrected for Age and Education? Insights From a Causal Perspective

**DOI:** 10.1093/aje/kwac159

**Published:** 2022-09-06

**Authors:** Marco Piccininni, Jessica L Rohmann, Maximilian Wechsung, Giancarlo Logroscino, Tobias Kurth

**Keywords:** age-education test correction, causal models, cognitive impairment, cognitive screening test, directed acyclic graphs

## Abstract

Cognitive screening tests such as the Mini-Mental State Examination are widely used in clinical routine to predict cognitive impairment. The raw test scores are often corrected for age and education, although documented poorer discrimination performance of corrected scores has challenged this practice. Nonetheless, test correction persists, perhaps due to the seemingly counterintuitive nature of the underlying problem. We used a causal framework to inform the long-standing debate from a more intuitive angle. We illustrate and quantify the consequences of applying the age-education correction of cognitive tests on discrimination performance. In an effort to bridge theory and practical implementation, we computed differences in discrimination performance under plausible causal scenarios using Open Access Series of Imaging Studies (OASIS)-1 data. We show that when age and education are causal risk factors for cognitive impairment and independently also affect the test score, correcting test scores for age and education removes meaningful information, thereby diminishing discrimination performance.

## Abbreviations

AUC: area under the receiver operating characteristic curve
DAG: directed acyclic graph
MMSE: Mini-Mental State Examination
OASIS: Open Access Series of Imaging Studies

Cognitive screening tests are tools used to screen for dementia and cognitive impairment (1, 2). These tests are validated by assessing their ability to predict clinical diagnoses, such as dementia and mild cognitive impairment, characterized by a decline in cognitive performance (1–6).

An individual’s raw test score is the numerical result of the test, such as the number of correct answers or errors, time to complete the test, or performance rating (5, 7). On their own, these raw scores are generally considered to have no inherent meaning and only become interpretable when compared with preexisting norms or standards from individuals with similar demographic characteristics (e.g., age, sex, ethnicity, and/or educational level) (5, 7). This comparison with the corresponding demographic-specific norm is conducted by transforming the raw score into a corrected score. In psychology, this practice is referred to as standardization, adjustment, or correction.

The correction of raw scores is often employed in cognitive screening (1, 8–11), despite having repeatedly encountered sharp criticism pertaining to its poor discrimination performance. First, theoretical concerns were raised together with initial empirical observations (12–19), and additional support was found in simulation studies (13).

However, the aforementioned criticism did not noticeably shift the paradigm. One reason might be the lack of an explicit causal framework, which, in our opinion, is necessary for an intuitive, complete understanding of this problem. Therefore, we aim to take a causal approach to address this long-standing debate regarding prediction performance. We provide an explanation for the counterintuitive results in the literature surrounding the problem of the age-education correction in cognitive screening. Specifically, we detail the explicit relationship between the correction of these tests and discrimination performance operationalized as area under the receiver operating characteristic curve (AUC).

## THEORETICAL BACKGROUND AND MAIN HYPOTHESIS

### What is test correction?

Test correction is a common practice that relies on normative data. For practical reasons, correction factors or norms are established using the raw scores of a normative sample, typically composed of so-called “healthy” individuals who are not affected by diseases thought to lead to cognitive or behavioral impairment (5, 10). We consider a normative sample to refer to a group of individuals without the specific condition (i.e., impairment) of interest. Once normative data are available for a given test, each new individual raw score is compared with the distribution of scores of individuals with similar demographic characteristics in the normative sample (5, 9). To make this comparison simpler and more explicit, the raw score is converted into a corrected score. The *z* score is the most commonly encountered example of correction in practice (5, 11, 20, 21), likely due to its several convenient statistical properties and straightforward interpretation (5).

The *z* score is the distance between an individual’s performance and the average performance of the group of individuals from the normative sample with similar demographics, measured in units of this group’s standard deviation (5, 7, 22). The *z*-score correction relies on the assumption that the distribution of normative data within each demographic stratum is approximately normal (5, 20). Alternatively, model-based corrections can be used to accommodate sparse data but rely on stronger assumptions (5, 11, 20, 21). Regardless of the correction method, the average score of all nonimpaired individuals in one demographic stratum will be the same as the average score of all nonimpaired individuals in any other demographic stratum. The correction process transforms the raw test scores into new corrected values by “removing” the components of the raw scores attributable to the demographic variables (22). This interpretation requires the assumption that the effects of demographic variables are the same among impaired and nonimpaired individuals.

Age and education are considered particularly important in cognitive assessment. Almost all tests for cognitive impairment are traditionally corrected for these 2 variables (23). This implies that the components of each raw score attributable to age and education are ignored when distinguishing between impaired and nonimpaired individuals. Only the comparison with the performance of the nonimpaired individuals within the same demographic stratum is considered. This means that an older individual with lower educational attainment having a low raw score may still be classified as “nonimpaired” or “normal” (17). Such an individual will be classified as “impaired” at a lower score than a younger person or one with a higher educational attainment (22).

The concept of age correction is thus intrinsically linked to the life-span development theoretical model (24), in which a certain amount of cognitive decline is considered normal, and the expected performance is a function of age (25). This stands in contrast with the biological aging model (26), in which any loss of functionality, even if attributable to aging, should be detected and potentially addressed in treatment strategies (25).

### Why can test correction be problematic?

The argument for accounting for demographic variables in cognitive screening tests is that they are known to have an impact on test performance (1, 5, 11, 27). Therefore, removing the components of the raw scores explained by these variables will reduce the variance of the distribution of the scores (5, 18). This reduces the “noise” and thereby makes the difference in test scores between impaired and nonimpaired individuals more pronounced, resulting in increased discrimination performance. Therefore, the use of such correction is often underpinned by observed associations between demographic variables and raw test scores (16).

Despite this seemingly sound rationale, a growing body of evidence challenges the case for correction (13). Already in 1996, the results from a large survey of older persons showed that the ability of a psychological screening battery to discriminate dementia cases was not improved by correcting for demographic characteristics compared with using the raw scores (15). Well-known screening tests, such as the Mini-Mental State Examination (MMSE) (16) and the modified 3MS version (12), when corrected, showed equal or worse performance compared with raw scores in screening for dementia and cognitive impairment. Furthermore, the ability of psychological tests to predict future progression to dementia was reduced by correction in longitudinal studies (17, 18). Moreover, using age-corrected test scores is known to lower the sensitivity for detecting mild cognitive impairment due to Alzheimer disease, especially outside the original development data sets (22).

The reason that the age correction reduces the discrimination performance is not due to a flawed rationale behind the correction but rather that it does not consider a crucial element of the whole picture. Indeed, age and education not only influence raw test scores, but they, themselves, are also causal risk factors for cognitive impairment and dementia.

Therefore, our hypothesis is that using the correction, which removes the impact of these variables from the raw scores, likely also removes some valuable predictive information provided by these risk factors, thereby diminishing the test’s ability to discriminate between impaired and nonimpaired individuals (12).

The general ideas that test correction ignores the relationships between demographic variables and the probability of being cognitively impaired and that the concept of using age-corrected scores to detect cognitive impairment can be misleading were first proposed by Berkman (19) and Sliwinski et al. (14, 22). Thereafter, O’Connell and Tuokko (13) illustrated how the correction differentially affects the overlap of the test score distribution of impaired and nonimpaired groups if the demographic variables only inject noise or are risk factors.

This theory was later corroborated in simulations and using real data (13). Overall, discrimination performance was found to be reduced when tests were corrected for demographic variables (13). After applying the correction, a lowered sensitivity was observed and, as a trade-off, a slight increase in specificity (13).

## CORRECTION FROM A CAUSAL PERSPECTIVE

Despite this empirical evidence and theoretical criticism, the practice of applying the age-education correction in cognitive screening persists. We argue that this is due to a perceived paradox pertaining to the problem at hand. First, theoretically, in different situations, the application of the same correction may be either beneficial or detrimental in terms of discrimination. Second, practically, it seems unintuitive that using additional information (i.e., about age and education) to create the corrected score ultimately results in an inferior discrimination performance.

Historically, many counterintuitive situations perceived to be paradoxes only became readily understandable in a causal framework (28). Although this age-education correction paradox arose in the domain of prediction, we argue it is no exception, as evidenced by the necessary use of causal terms (e.g., “impact,” “effect,” “attributable,” “explained,” and “risk factor”) to describe the problem. Indeed, recent work has shown that causal knowledge and causal diagrams can be used to inform prediction modeling strategies in biomedical research: specifically, to inform predictor selection, assess transportability, and predict outcomes under possible interventions (29–33). This work centers on the intersection of causal inference (focused on understanding determinants of and treatments for diseases) and prediction (focused on identifying clinical conditions to optimize care).

In the field of causal inference, directed acyclic graphs (DAGs) are used to visually depict underlying causal structures and inform strategies to address biases (28, 34, 35). In these causal diagrams, single nodes are used to represent variables, while arrows represent direct causal paths between 2 variables. DAGs are used to encode a priori assumptions behind the underlying data-generation process, and through a series of graphical rules, provide qualitative information about conditional independencies between the variables. Several helpful introductions to DAGs have been published elsewhere (28, 34–36).

### Understanding the age-education test correction using DAGs

**Figure 1:**
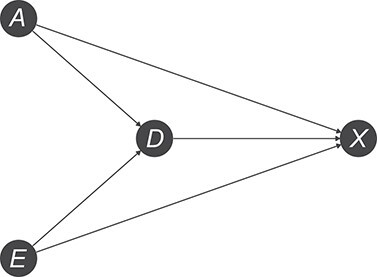
Directed acyclic graph depicting assumed causal relationships between age (*A*), educational level (*E*), cognitive impairment (*D*), and raw test score (*X*). Figure created using the R (R Foundation for Statistical Computing) package ggdag (51).

We can use a DAG to view the age-education correction problem through a causal lens. Specifically, we can draw a DAG describing the causal relationships between age (*A*), educational level (*E*), cognitive impairment (*D*), and raw test score (*X*). Age and education are known risk factors for cognitive impairment; therefore, we can draw an arrow from age (*A*) to cognitive impairment (*D*) and another arrow from education (*E*) to cognitive impairment (*D*) (Figure 1). Demographic factors are also known to be direct causes of cognitive test performance (*X*) (1, 5, 11, 27); for example, age and education may affect comprehension, education may affect cognitive reserve, and age affects the occurrence of other pathologies that can influence test performance. Moreover, the cognitive test performance (*X*) is also affected by cognitive impairment (*D*). These relationships are made explicit by the 3 arrows from *A*, *E*, and *D* into *X* (Figure 1).

Of course, this is a very parsimonious representation of reality, and the DAG could be expanded to include other causes of cognitive impairment and explicitly depict the measurement error in test performance; however, we believe this simple DAG is sufficient to correctly illustrate the problem at hand, which is also at the core of complex, real-world scenarios. Our DAG is implicitly supported by the intuition of Hessler et al. (17) and Magni et al. (27) (despite some inconsistencies in their reasoning) that age and education can be conceptualized as “confounding variables” of the relationship between raw cognitive screening test score and disease status.

In our DAG (Figure 1), 3 direct arrows connect node *D* to the other nodes, *A*, *E*, and *X*. This indicates that age, educational level, and raw test score each provide unique information about cognitive impairment that cannot be captured by the other 2 variables. In more technical terms, all 3 variables are part of *D*’s Markov blanket (37–39) and are needed for an optimal prediction in terms of calibration (29, 37, 39). Intuitively, it therefore seems wise to consider age, educational level, and the raw test score jointly to predict cognitive impairment. However, since the focus of our work is to inform the ongoing debate on the use of the age-education correction in cognitive screening, we compared only 2 commonly encountered strategies to predict cognitive impairment: 1) using the raw score alone or 2) using the corrected score alone.

There are 3 distinct paths connecting the cognitive impairment (*D*) node to the raw test score (*X*) node: $D\to X$, $D\leftarrow A\to X$, $D\leftarrow E\to X$. All of these paths are open; therefore, according to causal graph rules, information about cognitive impairment “flows” to the raw test score through all of them (28, 35, 36). This means that the association between raw test score and cognitive impairment observed in the data, given correct DAG specification and some other common assumptions (28, 35), is the joint result of these 3 paths. Of these 3 paths, only one ($D\to X$) is causal, while the paths passing through the nodes age or education are noncausal because they each include an arrow pointing from effect to cause.

The correction transforms the raw test scores by removing the components of the raw scores attributable to the demographic variables (22). Indeed, we can conceptualize the correction (under some assumptions presented explicitly later) as an attempt to remove the information flowing into *X* from *D* via the 2 noncausal paths, specifically through the arrows $A\to X$ and $E\to X$. If we assume the DAG in Figure 1 is correct, then deleting the information delivered by the arrows $A\to X$ and $E\to X$ will modify the association between *X* and *D* because these 2 arrows each constitute the final step in a path that delivers information about cognitive impairment to the raw test score. Therefore, the age-education correction implies a loss of useful information assuming this causal structure. In the next section, we show that given the signs of the causal effect sizes in play, this loss of information translates into a lower discrimination performance.

**Figure 2:**
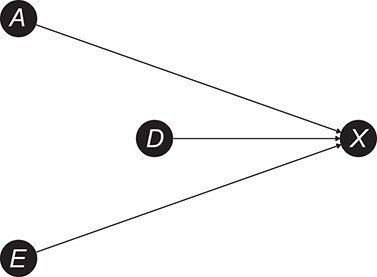
Directed acyclic graph depicting assumed causal relationships between age (*A*), educational level (*E*), cognitive impairment (*D*), and raw test score (*X*) in the scenario in which age and education are assumed not to be risk factors for cognitive impairment. Figure created using the R (R Foundation for Statistical Computing) package ggdag (51).

Ultimately, the consequence of this action depends on whether age and education are risk factors for cognitive impairment. If one alternatively assumes that age and education are not causes of cognitive impairment (see modified DAG in Figure 2), neither age nor education are on a path through which information about cognitive impairment flows to raw test score. In this scenario, the arrows $A\to X$ and $E\to X$ deliver no information about cognitive impairment (*D*) to the raw test score (*X*), and simply represent injected noise. In such a situation, the correction is actually beneficial, since it removes this noise.

### The age-education correction in a fully specified causal model

Returning to the DAG depicted in Figure 1, each node represents a variable and the diagram represents an underlying structural model (28, 35, 36). Each variable can be written as the output of an assignment function of the variable’s direct causes (parent nodes) as well as some unknown random noise variable (28, 35, 36). The assignment formalizes the directionality of the corresponding causal relationships. Noise variables are not conventionally depicted in DAGs and are assumed to be independent of each other.

Suppose our causal model is fully specified and we know both the true assignment functions and the noise probability distributions. For individuals $i=1,\dots, n$, we model age and education as random variables ${A}_i$ and ${E}_i$ with distributions ${P}^A$ and ${P}^E$, respectively. A Bernoulli variable ${D}_i$ with success probability ${p}_i$ denotes an individual’s cognitive status, with ${D}_i=1$ denoting cognitive impairment. The success probability is assigned as a function of its parents’ nodes. We define

$$ \begin{align*}{p}_i=\frac{\exp\ \big({\gamma}_0+{\gamma}_1{A}_i+{\gamma}_2{E}_i\big)}{1+\exp\ \big({\gamma}_0+{\gamma}_1{A}_i+{\gamma}_2{E}_i\big)}.\end{align*}$$

We choose ${\gamma}_1\ge 0$ and ${\gamma}_2\le 0$ to acknowledge that higher age is a known risk factor for cognitive impairment while higher education is known to protect against it.

For an individual’s raw test score, we assume a simple linear assignment function. Let $ \big\{{\varepsilon}_i:i=1,\dots, n\big\}$ be independent and normally distributed with mean zero and positive variance $ {\sigma}^2 $. Then

$$ \begin{align*}{X}_i={\beta}_0+{\beta}_1\ {A}_i+{\beta}_2\ {E}_i+{\beta}_3\ {D}_i+{\varepsilon}_i,\end{align*}$$

where ${\beta}_1,{\beta}_3\le 0$ and ${\beta}_2\ge 0$. As such, we assume the mean raw test score decreases with age, increases with education, and decreases with cognitive impairment. These effects have been reported for several cognitive screening tests (1, 11, 13, 27) such as the MMSE. Here, we assume no interaction term in the assignment function. These assumptions are of course idealizing, particularly the normal distribution assumption, common in many model-based methods for test correction (11, 20, 21), which allows us to conveniently ignore ceiling and floor effects.

The corrected score (${Z}_i$) of an individual with age ${a}_i$ and educational level ${e}_i$ is obtained by subtracting the expected raw score of nonimpaired individuals with the same age and education from the individual’s raw score, and then dividing by the corresponding conditional standard deviation,

$$ \begin{align*}{Z}_i=\frac{X_i-\big({\beta}_0+{\beta}_1\ {a}_i+{\beta}_2\ {e}_i\big)}{\sigma }.\end{align*}$$

To compare discrimination performance, we used the difference between the AUC of the raw score, $\mathrm{AUC}(X)$, and the AUC of the corrected score, $\mathrm{AUC}(Z)$. The choice of this metric is consistent with previously published simulation studies (13). It is known that the AUC is the probability that a randomly drawn individual from the impaired group has a lower raw score compared with a randomly drawn individual from the nonimpaired group. Therefore,

$$ \begin{align*}\mathrm{AUC}(X)&=\Pr \{{X}_1< {X}_0\big|{D}_i=i\}\\ &{}=\Pr \{{\beta}_1\ ({A}_1-{A}_0)+{\beta}_2\ ({E}_1-{E}_0)\\&\qquad+{\beta}_3< {\varepsilon}_0-{\varepsilon}_1\big|{D}_i=i\}\\ &{}=\textstyle\int \Pr \{{\beta}_1\ ({a}_1-{a}_0)+{\beta}_2\ ({e}_1-{e}_0)\\&\qquad+{\beta}_3< {\varepsilon}_0-{\varepsilon}_1\big|{E}_i={e}_i,{A}_i={a}_i,{D}_i=i\}\\ &\qquad\qquad\qquad{P}^{A_1,{A}_0,{E}_1,{E}_0\mid {D}_i=i}({da}_1,{da}_0,{de}_1,{de}_0),\end{align*}$$

where conditions of the form ${A}_i={a}_i,{D}_i=i$ are to be understood as ${A}_1={a}_1,{A}_0={a}_0,{D}_1=1,{D}_0=0$. Since the noise variables are independent standard normal variables, their difference is also normal with mean zero and variance $2{\sigma}^2$. Furthermore, they are independent of all the other variables. Hence, the probability inside the integral can be written as $1-{\Phi}_{0,2{\sigma}^2}({\beta}_1\ ({a}_1-{a}_0)+{\beta}_2\ ({e}_1-{e}_0)+{\beta}_3)$. Following the same rationale, we see that:

$$ \begin{align*}\mathrm{AUC}(Z)&=\Pr \{{Z}_1 < {Z}_0\big|{D}_i=i \}\\ &{}=\Pr \{{X}_1- ({\beta}_0+{\beta}_1\ {A}_1+{\beta}_2\ {E}_1)< {X}_0\\&\qquad-({\beta}_0+{\beta}_1\ {A}_0+{\beta}_2\ {E}_0)\big|{D}_i=i\}\\ &{}=\Pr \{{\beta}_3< {\varepsilon}_0-{\varepsilon}_1\big|{D}_i=i\}\\ &{}=1-{\Phi}_{0,2{\sigma}^2}({\beta}_3).\end{align*}$$

A detailed mathematical derivation of $\mathrm{AUC}(X)$ is provided in Web Appendix 1 (available at https://doi.org/10.1093/aje/kwacc159). These mathematical expressions corroborate the intuitive reasoning we outlined in the previous section. The discrimination performance of the corrected test score ($\mathrm{AUC}(Z)$) depends only on the direct causal effect (${\beta}_3$) of cognitive impairment on the raw test score. This is in line with our previous statement that the corrected score uses only information flowing through the direct arrow $D\to X$ as the information about cognitive impairment provided by the paths containing age or education is removed.

By contrast, $\mathrm{AUC}(X)$ depends additionally on ${\gamma}_1,{\gamma}_2$ and ${\beta}_1,{\beta}_2$ (i.e., on the strength of the conditional causal relationships between the demographic factors and cognitive impairment on one hand, and between the demographic factors and the raw test score on the other; see Web Appendix 1). Thus, the choice whether to apply the correction must be informed by knowledge about the underlying causal relationships. Indeed, the difference between the discrimination performance of the 2 approaches hinges on the magnitude of all these causal effects. Although the derived expressions are too complicated for the analytical computation of $\mathrm{AUC}(X)-\mathrm{AUC}(Z)$, below we present a case study to illustrate its dependence on the coefficients ${\gamma}_1,{\gamma}_2,{\beta}_1,{\beta}_2$, and ${\beta}_3$ using numerical analysis.

**Figure 3:**
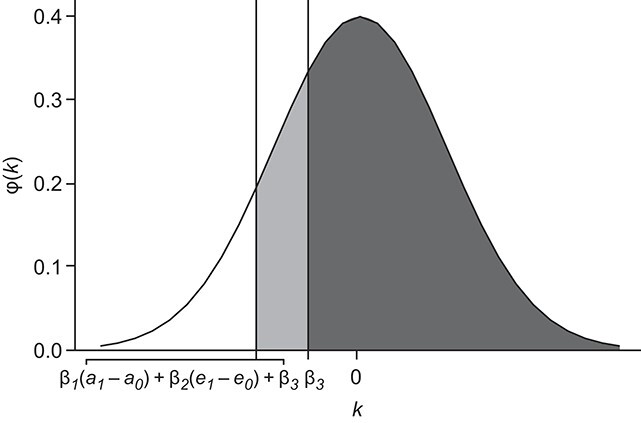
Plot representing the generic density function $\varphi (k)$ of a normal distribution centered at zero. The area below the curve provides a geometrical intuition of the difference between the area under the receiver operating characteristic curve (AUC) of the corrected score (AUC(*Z*); black area) and the one of the raw test score (AUC(*X*); gray and black areas).

Informally, we can already anticipate the consequence of using the age-education correction on the AUC if age and education affect both cognitive impairment (${\gamma}_1>0$ and ${\gamma}_2<0$) and test score (${\beta}_1<0$ and ${\beta}_2>0$). We saw that $\mathrm{AUC}(Z)$ corresponds to the probability of drawing a value higher than ${\beta}_3$ from a normal distribution with mean 0 and variance ${\sigma}^2$, depicted as the black area in Figure 3. Similarly, $\mathrm{AUC}(X)$ corresponds to the probability of drawing a value higher than ${\beta}_1\ ({A}_1-{A}_0)+{\beta}_2\ ({E}_1-{E}_0)+{\beta}_3$ from the same normal distribution. Since ${A}_1$, ${A}_0$, ${E}_1$, and ${E}_0$ are random variables, $\mathrm{AUC}(X)$ actually corresponds to a weighted average of the probabilities of this event for the different realizations of ${A}_1$, ${A}_0$, ${E}_1$, and ${E}_0$. For simplicity, however, let’s imagine that $\mathrm{AUC}(X)$ is the area under the normal distribution to the right of the “typical” realization ${\beta}_1\ ({a}_1-{a}_0)+{\beta}_2\ ({e}_1-{e}_0)+{\beta}_3$ (gray and black areas in Figure 3). If age and education are risk factors for cognitive impairment, we generally expect that ${a}_1>{a}_0$ and ${e}_1<{e}_0$ (i.e., individuals with cognitive impairment have higher age and lower education compared with individuals without cognitive impairment). In this scenario, since ${\beta}_1<0$ and ${\beta}_2>0$, the $\mathrm{AUC}$ of the raw score will generally be higher than the one of the corrected score (Figure 3). Notably, the same reasoning also applies if the effects of age and education on the test score are different among individuals with and without impairment (see Web Appendix 2).

## CASE STUDY USING OASIS-1 DATA

To compare the discrimination performance of the raw versus the corrected score of a cognitive screening test, we computed $\mathrm{AUC}(X)$ and $\mathrm{AUC}(Z)$ under plausible parameter constellations using data from the Open Access Series of Imaging Studies (OASIS) (40). The OASIS project makes neuroimaging data sets freely available to the scientific community. We used the data from OASIS-1, a cross-sectional collection of information about brain magnetic resonance imaging of 416 right-handed individuals ranging in age from 18 to 96 years (41). Young and middle-aged adults were recruited from the Washington University community, while older individuals were recruited from a longitudinal pool of the Washington University Alzheimer Disease Research Center (41). We considered only the 198 individuals aged 60 or older who underwent a full clinical assessment. Full details are available elsewhere (41).

We extracted the values of age, educational level, Clinical Dementia Rating (CDR) scale, and MMSE raw test score for each individual. For the purposes of this example, we converted the categorical educational level variable into number of years of education (less than high school graduate: 8 years; high school graduate: 12 years; some college: 14 years; college graduate: 16 years; beyond college: 18 years) (42).

We defined the outcome of interest (cognitive impairment) as having a CDR greater than zero (outcome prevalence: 50.50%). We considered this data suitable to generate parameters for our illustrative purposes because no cognitive test information was used to define cognitive impairment. Instead, it was exclusively based on clinical assessment using the CDR scale (41).

For simplicity, we assumed age to be normally distributed and the number of years of education to follow a χ^2^ distribution. We estimated the mean age (76.343) and standard deviation (8.087) and the mean number of years of education (13.848) from the OASIS-1 data set. The linear and logistic regression parameter estimation yielded ${\beta}_0=26.785,{\beta}_1=-0.011$ (effect of age on test score), ${\beta}_2=0.209$ (effect of education on test score), ${\beta}_3=-4.415$ (effect of cognitive impairment on test score), $\sigma =3.028$ and ${\gamma}_0=0.707,{\gamma}_1=0.011$ (natural logarithm of the odds ratio for conditional effect of age on cognitive impairment), and ${\gamma}_2=-0.113$ (natural logarithm of the odds ratio for conditional effect of education on cognitive impairment).

We then estimated $\mathrm{AUC}(X)$ for the MMSE raw test score using the R (R Foundation for Statistical Computing, Vienna, Austria) function hcubature() (43) to solve the quadruple integral (Web Appendix 3), additionally relying on a wrapper accommodating infinite boundaries (44). $\mathrm{AUC}(Z)$ was computed using the R function pnorm().

We computed the difference between the AUCs of the raw and corrected test scores for all possible combinations of the following parameter values, including the OASIS-1-derived estimates: ${\gamma}_1=(0,0.011,0.05,0.1)$; ${\gamma}_2=(-0.3,-0.113,0)$; ${\beta}_1=(-0.1,-0.05,-0.011,0)$; ${\beta}_2=(0,0.209,0.4)$; ${\beta}_3=(-7,-4.415)$. In all scenarios, we held the parameters ${\gamma}_0$ and $\sigma$ constant at the values estimated from the OASIS-1 data set. Given correct specifications of the DAG, equations, and noise functions, Web Figure 1 illustrates the difference in discrimination performance of these 2 approaches under different causal scenarios.

For the scenario in which the OASIS-1-derived parameters were used (black square in Web Figure 1), the AUC of the corrected score was 0.017 lower than the AUC of the raw score, indicating poorer discrimination performance.

If there is no direct causal effect of age and education on the raw test score (imagine the DAG in Figure 1 without $A\to X$ and $E\to X$), meaning ${\beta}_1,{\beta}_2=0$, it becomes irrelevant whether the correction is used, because there is no difference in the discrimination performance of the corrected and the raw scores (bottom row in Web Figure 1).

In each outlined quadrant, moving toward the top-right corner, the coloration becomes increasingly red. This means that, holding the other parameters constant, the correction becomes increasingly detrimental as ${\gamma}_1$ increases and ${\beta}_1$ decreases. This corresponds to an increase in the magnitude of the conditional direct effects of age on cognitive impairment ($A\to D$) and age on test performance ($A\to X$). Therefore, the information flowing from *D* to *X* through the path $D\leftarrow A\to X$ also increases (Figure 1), and the correction therefore results in a greater information loss. The same argument holds for the other noncausal path $ D\leftarrow E\to X$. Holding all other parameters constant, the correction results in increasingly poor discrimination performance as $ {\gamma}_2 $ decreases and $ {\beta}_2 $ increases. This is reflected in Web Figure 1: Holding $ {\beta}_3 $ constant, moving toward the top-right corner, the coloration of the quadrant becomes more red. Under these circumstances, by preventing flow from E to X, the correction results in a larger loss of information about cognitive impairment delivered via the path $ D\leftarrow E\to X $

Conversely, the correction appears beneficial if ${\gamma}_1,{\gamma}_2=0$ and ${\beta}_1,{\beta}_2\ne 0$. Indeed, if age and education do not causally affect cognitive impairment (Figure 2), they only inject noise into the raw test score. As always, the correction corresponds to removing information delivered by the arrows $A\to X$ and $E\to X$, but this time the information is just noise (*E* and *A* are not on an open path between *D* and *X*). Therefore, correction results in a reduction of this noise and a higher discrimination performance.

If only one of the demographic variables (i.e., age or educational level) is a cause of cognitive impairment, applying the age-education correction may result in either a lower or higher discrimination performance compared with the raw score. This ultimately depends on the trade-off between the amounts of useful information lost and noise removed by correction.

Let us now focus on the relationship between cognitive impairment and raw test score ($D\to X$ in Figure 1). Holding all other parameters constant, as the causal effect of cognitive impairment on test score becomes larger in magnitude (lower ${\beta}_3$), more information flows through the path $D\to X$ compared with the 2 other paths into *X*. In this case, the decision whether or not to correct becomes less relevant because any potential harms or benefits of the correction are attenuated.

In using OASIS-1 data to estimate some plausible parameters in this illustrative example, we acknowledge that some distributional and functional assumptions are unrealistic (e.g., linearity, no interaction terms, homoscedasticity, normality of errors, and absence of ceiling and floor effects in the test score). Moreover, in the OASIS-1 data set, ${\beta}_1$ and ${\gamma}_1$ were very small. We emphasize that the use of these parameter estimates was only a starting point and has allowed us to readily show the impact of correction on discrimination performance under different causal scenarios, bridging the theoretical causal model and a practical implementation. We hope this simplified, didactic exercise serves as a basis for further empirical confirmation.

## CONCLUSION

Age-education correction of neuropsychological and cognitive screening test scores is a common practice in the context of neurodegenerative diseases. Corrected scores are anchored in numerous definitions of mild cognitive impairment held in high esteem (45). Both the original mild cognitive impairment criteria proposed by Petersen et al. (46, 47) and the more recent *Diagnostic and Statistical Manual of Mental Disorders* (5th edition) definition of mild neurocognitive disorder (48) rely on the comparison of test scores with appropriate norms (defined by age alone or age and education) to make a diagnosis.

Although initial criticisms of test correction have been described in the literature, the problem of poorer discrimination performance has been largely ignored by the clinical and research communities. One explanation may be that in the absence of an explicit causal framework, the problem is far from intuitive.

We approached the problem from a causal perspective, using DAGs to depict underlying data-generation processes and graph rules to intuitively show the consequences of correcting cognitive screening tests for age and education. Assuming a fully-specified causal model, we compared the discrimination performance of the raw and corrected test scores in terms of AUC for a plausible range of causal scenarios.

If age and education are causal risk factors for cognitive impairment, then the raw score contains information about cognitive impairment also delivered by age and education via 2 noncausal paths. As we showed, applying the age-education correction in this causal scenario removes useful information and results in worse discrimination performance compared with using the raw test score given the signs of the causal effect sizes in play.

Since age and education are well-known causal risk factors for cognitive impairment, we argue that the age-education correction is generally hard to justify in real-world scenarios. Thus, we believe correction should not be performed without careful consideration of the underlying causal processes, especially considering the importance of this prediction task in clinical practice. While our focus is on cognitive screening tests, our results are more generally applicable to all situations in which the same underlying causal structure is realistic. We believe that our conclusion about discrimination performance holds for any single neuropsychological test used to predict cognitive impairment, given the conceptual similarity (6), although it is unlikely that a single neuropsychological test is used to predict the cognitive status in practice. Furthermore, our work provides insights on how to better predict cognitive impairment when trying to provide clinical guidance, and our results do not apply in the setting of causal research on the determinants of cognitive impairment.

In this work, we focused on discrimination performance and the question of whether the age-education correction should be applied. However, potential implications of this correction for other key prediction metrics (i.e., calibration), predictor selection strategies, and transportability should also be explored. Another important aspect to consider is societal impact. Using neuropsychological test scores without age-education correction results in a higher likelihood of classifying individuals who are older and less educated as cognitively impaired. This may exacerbate or attenuate social inequalities depending on the specific societal context, including the vulnerability of these sociodemographic groups, societal stigma, access to care, and access to financial support. For instance, it has been shown in the context of racial discrimination in workers’ compensation how demographic correction can either amplify or diminish inequalities depending on broader societal conditions (49). Furthermore, the statistical tool of correction is directly linked to the technique proposed to mitigate algorithmic bias and achieve so-called counterfactual fairness (50).

We believe our work provides important evidence on how causal knowledge expressed as a parsimonious representation of the underlying data-generation process is useful to address relevant prediction tasks regularly encountered in clinical practice.

## CODE AVAILABILITY

Web Appendix 3 contains the R (R Foundation for Statistical Computing) code for 1) estimating the OASIS-1 derived parameters, 2) building the function to approximate the AUC for raw and corrected test scores under all described scenarios, and 3) approximating the AUC for raw and corrected test scores in the scenario defined by the combination of parameters estimated from OASIS-1 data.

## Supplementary Material

Web_Material_kwac159
