# Supplementary material for: Should Cognitive Screening Tests Be Corrected for Age and Education? Insights From a Causal Perspective
Source: Am J Epidemiol. 2022 Sep 6;192(1):93–101. doi: 10.1093/aje/kwac159 (PMC9825732; doi:10.1093/aje/kwac159)
Supplement: Web_Material_kwac159 [file web_material_kwac159.pdf]

Web Material

Should cognitive screening tests be corrected for age and education? Insights from a causal perspective

Marco Piccininni, Jessica L. Rohmann, Maximilian Wechsung, Giancarlo Logroscino, Tobias Kurth

Contents

Web Appendix 1 2

Web Appendix 2 3

Web Appendix 3 4

R code sample (R v4.0.3, RStudio v1.1.456) 4

Web Figure 1 6

Web Appendix 1

$$\begin{aligned}
&\mathrm{AUC}(X)\\
&= \Pr\big\{X_1 < X_0 \bigm| D_i = i\big\}\\
&= \Pr\big\{\beta_1\,(A_1 - A_0) + \beta_2\,(E_1 - E_0) + \beta_3 < \varepsilon_0 - \varepsilon_1 \bigm| D_i = i\big\}\\
&= \int \Pr\big\{\beta_1\,(a_1 - a_0) + \beta_2\,(e_1 - e_0) + \beta_3 < \varepsilon_0 - \varepsilon_1 \bigm| E_i = e_i,\, A_i = a_i,\, D_i = i\big\}\\
&\qquad\qquad\qquad\qquad\qquad\qquad P^{A_1,A_0,E_1,E_0|D_i=i}(\mathrm{d}a_1, \mathrm{d}a_0, \mathrm{d}e_1, \mathrm{d}e_0)\,,
\end{aligned}$$

where conditions of the form $A_i = a_i$, $D_i = i$ are to be understood as $A_1 = a_1$, $A_0 = a_0\,, D_1 = 1$, $D_0 = 0$. Since the noise variables are independent standard normal variables, their difference is also normal with mean zero and variance $2\sigma^2$. Furthermore, they are independent of all the other variables. Hence, the probability inside the integral can be written as $1 - \Phi_{0,2\sigma^2}\big(\beta_1\,(a_1 - a_0) + \beta_2\,(e_1 - e_0) + \beta_3\big)$.

Since all individuals are mutually independent, $P^{A_1,A_0,E_1,E_0|D_i=i} = P^{A_0,\,E_0|D_0=0} \otimes P^{A_1,\,E_1|D_1=1}$. Moreover,

$$\frac{\mathrm{d}P^{A_1,\,E_1|D_1=1}}{\mathrm{d}P^{A_1,\,E_1}} = \frac{\Pr\{D_1 = 1 \mid A_1 = a_1,\, E_1 = e_1\}}{\int \Pr\{D_1 = 1 \mid A_1 = a_1,\, E_1 = e_1\}\; P^{A_1,\,E_1}(\mathrm{d}a_1, \mathrm{d}e_1)}$$

almost surely. An according equality holds for $P^{A_0,\,E_0|D_0=0} \big/ \mathrm{d}P^{A_0,\,E_0}$. Furthermore, because of the independence entailed in the DAG of Figure 1, we have $P^{A,E} = P^A \otimes P^E$. Hence, we can write

$$\begin{aligned}
&\mathrm{AUC}(X)\\
&= \{C(1-C)\}^{-1} \int \big\{1 - \Phi_{0,2\sigma^2}\big(\beta_1\,(a_1 - a_0) + \beta_2\,(e_1 - e_0) + \beta_3\big)\big\}\\
&\frac{\exp\big(\gamma_0 + \gamma_1\,a_1 + \gamma_2\,e_1\big)}{\big\{1 + \exp(\gamma_0 + \gamma_1\,a_1 + \gamma_2\,e_1)\big\}\big\{1 + \exp(\gamma_0 + \gamma_1\,a_0 + \gamma_2\,e_0)\big\}}\; P^A(\mathrm{d}a_0)P^E(\mathrm{d}e_0)P^A(\mathrm{d}a_1)P^E(\mathrm{d}e_1)\,,
\end{aligned}$$

where $C = \int \frac{\exp(\gamma_0 + \gamma_1\,a + \gamma_2\,e)}{1 + \exp(\gamma_0 + \gamma_1\,a + \gamma_2\,e)}\; P^A(\mathrm{d}a)P^E(\mathrm{d}e)$. Following the same rationale, we see that

$$\begin{aligned}
\mathrm{AUC}(Z) &= \Pr\big\{Z_1 < Z_0 \bigm| D_i = i\big\}\\
&= \Pr\big\{X_1 - \big(\beta_0 + \beta_1\,A_1 + \beta_2\,E_1\big) < X_0 - \big(\beta_0 + \beta_1\,A_0 + \beta_2\,E_0\big) \bigm| D_i = i\big\}\\
&= \Pr\big\{\beta_3 < \varepsilon_0 - \varepsilon_1 \bigm| D_i = i\big\}\\
&= 1 - \Phi_{0,2\sigma^2}(\beta_3)\,.
\end{aligned}$$

Web Appendix 2

Let's assume that:

$$X_i = \beta_0 + \beta_1 A_i + \beta_2 E_i + \beta_3 D_i + \beta_4 D_i A_i + \beta_5 D_i E_i + \varepsilon_i$$

where $\beta_1, \beta_3 \leq 0$ and $\beta_2 \geq 0$.

$$\begin{aligned}
&\text{AUC}(X)\\
&= \Pr\big\{X_1 < X_0 \bigm| D_i = i\big\}\\
&= \Pr\big\{\beta_1 (A_1 - A_0) + \beta_2 (E_1 - E_0) + \beta_3 + \beta_4 A_1 + \beta_5 E_1 < \varepsilon_0 - \varepsilon_1 \bigm| D_i = i\big\}\\
&= \int \Pr\big\{\beta_1 (a_1 - a_0) + \beta_2 (e_1 - e_0) + \beta_3 + \beta_4 a_1 + \beta_5 e_1 < \varepsilon_0 - \varepsilon_1 \bigm| E_i = e_i,\, A_i = a_i,\, D_i = i\big\}\\
&\qquad\qquad P^{A_1, A_0, E_1, E_0 | D_i = i}(\mathrm{d}a_1, \mathrm{d}a_0, \mathrm{d}e_1, \mathrm{d}e_0)\,,
\end{aligned}$$

As above, the probability inside the integral can be written as $1 - \Phi_{0,2\sigma^2}\big(\beta_1 (a_1 - a_0) + \beta_2 (e_1 - e_0) + \beta_3 + \beta_4 a_1 + \beta_5 e_1\big)$. And relying on the same reasoning outlined for the simple case, we can write:

$$\begin{aligned}
\text{AUC}(X) = \{C(1-C)\}^{-1} \int \big\{1 - \Phi_{0,2\sigma^2}\big(\beta_1 (a_1 - a_0) + \beta_2 (e_1 - e_0) + \beta_3 + \beta_4 a_1 + \beta_5 e_1\big)\big\}\\
\frac{\exp\big(\gamma_0 + \gamma_1 a_1 + \gamma_2 e_1\big)}{\big\{1 + \exp(\gamma_0 + \gamma_1 a_1 + \gamma_2 e_1)\big\}\big\{1 + \exp(\gamma_0 + \gamma_1 a_0 + \gamma_2 e_0)\big\}}\ P^A(\mathrm{d}a_0) P^E(\mathrm{d}e_0) P^A(\mathrm{d}a_1) P^E(\mathrm{d}e_1)\,,
\end{aligned}$$

where $C = \int \frac{\exp(\gamma_0 + \gamma_1 a + \gamma_2 e)}{1 + \exp(\gamma_0 + \gamma_1 a + \gamma_2 e)}\ P^A(\mathrm{d}a) P^E(\mathrm{d}e)$. Following the same rationale, we see that

$$\begin{aligned}
\text{AUC}(Z) &= \Pr\big\{Z_1 < Z_0 \bigm| D_i = i\big\}\\
&= \Pr\big\{X_1 - \big(\beta_0 + \beta_1 A_1 + \beta_2 E_1\big) < X_0 - \big(\beta_0 + \beta_1 A_0 + \beta_2 E_0\big) \bigm| D_i = i\big\}\\
&= \Pr\big\{\beta_3 + \beta_4 A_1 + \beta_5 E_1 < \varepsilon_0 - \varepsilon_1 \bigm| D_1 = 1\big\}\\
&= \int \Pr\big\{\beta_3 + \beta_4 a_1 + \beta_5 e_1 < \varepsilon_0 - \varepsilon_1 \bigm| E_1 = e_1,\, A_1 = a_1,\, D_1 = 1\big\} P^{A_1, E_1 | D_1 = 1}(\mathrm{d}a_1, \mathrm{d}e_1)\\
&= C^{-1} \int \big\{1 - \Phi_{0,2\sigma^2}\big(\beta_3 + \beta_4 a_1 + \beta_5 e_1\big)\big\} \frac{\exp\big(\gamma_0 + \gamma_1 a_1 + \gamma_2 e_1\big)}{\big\{1 + \exp(\gamma_0 + \gamma_1 a_1 + \gamma_2 e_1)\big\}}\ P^A(\mathrm{d}a_1) P^E(\mathrm{d}e_1)
\end{aligned}$$

Web Appendix 3

R code sample (R v4.0.3, RStudio v1.1.456)

Packages and functions

```
library(tidyverse)
library(cubature)

hcubature.inf <- function() {
  cl <- match.call()
  cl[[1L]] <- quote(cubature::hcubature)
  if(all(is.finite(c(lowerLimit,upperLimit)))) return(eval.parent(cl))
  cl[['upperLimit']] <- atan(upperLimit)
  cl[['lowerLimit']] <- atan(lowerLimit)
  f <- match.fun(f)
  cl[['f']] <- if(!vectorInterface)
    function(x, ...) f(tan(x), ...) / prod(cos(x))^2
  else
    function(x, ...) f(tan(x), ...) / rep(apply(cos(x), 2, prod)^2, each=fDim)
  eval.parent(cl)
}
formals(hcubature.inf) <- formals(cubature::hcubature)
```

The hcubature.inf wrapper function was provided by Simen Gaure and is available **here**

Import and transform OASIS-1 data

Import the data, exclude individuals with age lower than 60, create the variable cognitive impairment (D), and convert education categories in years of education.

```
dati <- read_csv("oasis/oasis_cross-sectional.csv") %>% filter(Age>=60) %>%
        mutate(D=CDR>0,
              Educ_y=case_when(Educ==1~ 8,
                              Educ==2~ 12,
                              Educ==3~ 14,
                              Educ==4~ 16,
                              Educ==5~ 18))
```

Estimate parameters of interest

```
oasis_mean_age <- mean(dati$Age)
oasis_sd_age <- sd(dati$Age)
oasis_mean_educ <- mean(dati$Educ_y)
oasis_mod_D <- dati %>% glm(D ~ Age + Educ_y, data=. , family=binomial(link="logit"))
oasis_gamma0 <- coefficients(oasis_mod_D)[1]
oasis_gamma1 <- coefficients(oasis_mod_D)[2]
oasis_gamma2 <- coefficients(oasis_mod_D)[3]
oasis_mod_x <- dati  %>%  lm(MMSE ~ Age + Educ_y + D, data=.)
```

```
oasis_beta0 <- coefficients(oasis_mod_x)[1]
oasis_beta1 <- coefficients(oasis_mod_x)[2]
oasis_beta2 <- coefficients(oasis_mod_x)[3]
oasis_beta3 <- coefficients(oasis_mod_x)[4]
oasis_sigma <- summary(oasis_mod_x)$sigma
```

Approximate AUC(X), AUC(Z), and their difference

```
fun_diff <-
function(mean_age, sd_age, mean_educ, gamma0, gamma1, gamma2, beta1, beta2, beta3, sigma){

f_C <- function(x) {
   a1 <- x[1]
   e1 <- x[2]
   boot::inv.logit(gamma0 +gamma1*a1+gamma2*e1) * dnorm(a1,mean_age,sd_age) *
   dchisq(e1,mean_educ)}
C <- hcubature.inf(f_C, c(-Inf, 0), c(Inf, Inf))$integral

f <- function(x) {
   a1 <- x[1]
   a0 <- x[2]
   e1 <- x[3]
   e0 <- x[4]
   (1-pnorm(beta1*(a1-a0)+beta2*(e1-e0)+beta3,0,sqrt(2*sigma^2))) *
     boot::inv.logit(gamma0 +gamma1*a1+gamma2*e1) * dnorm(a1,mean_age,sd_age) *
     dchisq(e1,mean_educ) * ((1+exp(gamma0 +gamma1*a0+gamma2*e0))^(-1)) *
     dnorm(a0,mean_age,sd_age) * dchisq(e0,mean_educ) / (C*(1-C))}

AUC_x_f <- hcubature.inf(f, c(-Inf, -Inf, 0, 0), c(Inf, Inf, Inf, Inf))$integral
AUC_z_f <- unname(1 - pnorm(beta3,0,sqrt(2*sigma^2)))

return(list=c(AUC_x=AUC_x_f,AUC_z=AUC_z_f,diff=AUC_x_f-AUC_z_f))
}
```

Example using OASIS-1 derived parameter estimates

```
fun_diff(oasis_mean_age, oasis_sd_age, oasis_mean_educ, oasis_gamma0, oasis_gamma1,
         oasis_gamma2, oasis_beta1, oasis_beta2, oasis_beta3, oasis_sigma)
```

Web Figure 1

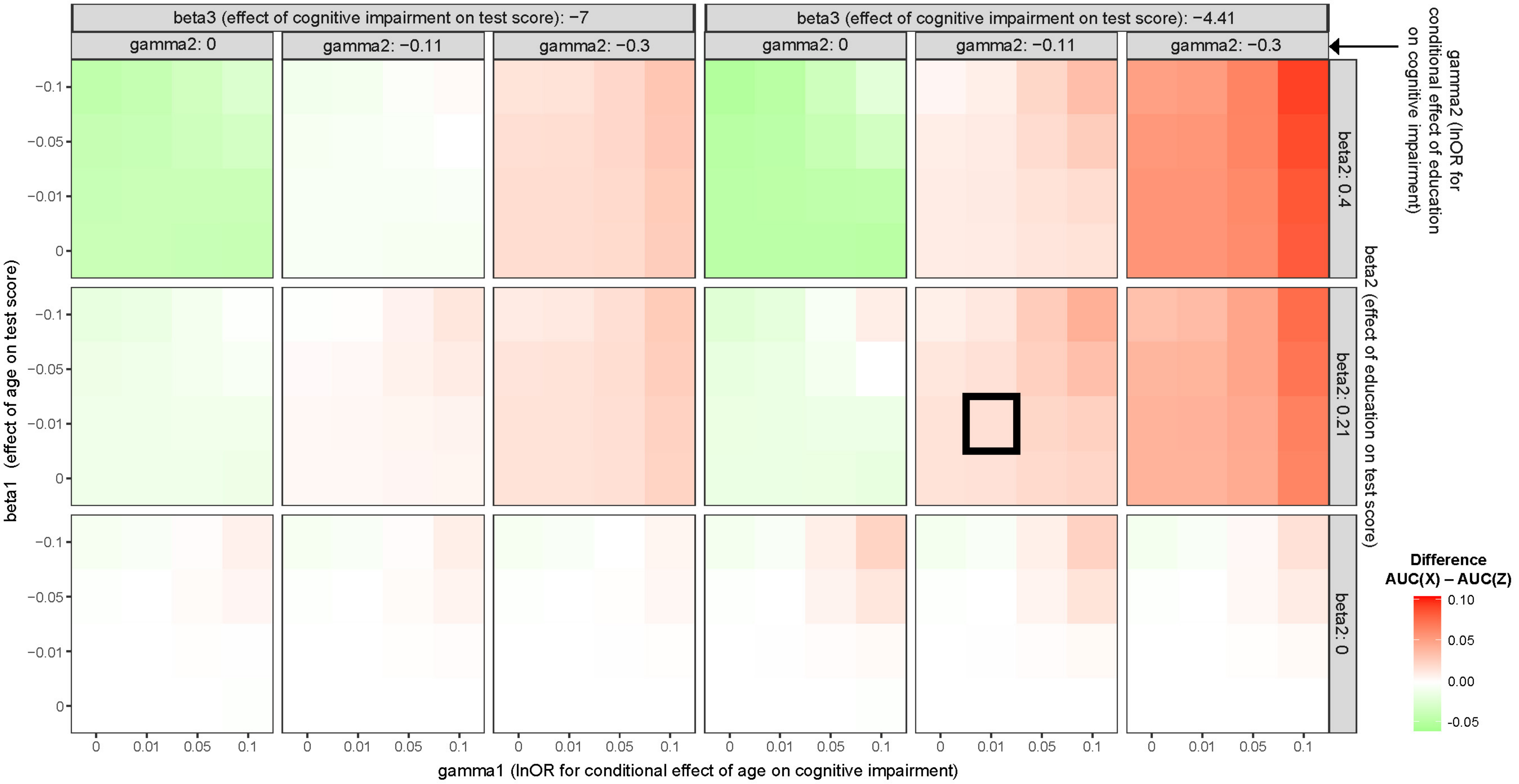

Web Figure 1. Differences between the area under the curve for the raw test score, AUC(X), and for the corrected test score, AUC(Z), based on different parameters representing various underlying causal scenarios. γ_1 (gamma1) and γ_2 (gamma2) represent the conditional causal effect of age and education on cognitive impairment expressed as logarithms of the odds ratio (lnOR). β_1 (beta1), β_2 (beta2), and β_3 (beta3) represent the causal effects of age, education, and cognitive impairment on the test performance. The black square indicates the combination of parameters estimated using OASIS-1 data. Increasing saturation of red color represents increasingly detrimental impact of age-education test correction on discrimination performance. In contrast, increasing green saturation indicates more beneficial impact of age-education test correction on discrimination performance. In all scenarios, other parameters were fixed at the values estimated from OASIS-1 data: age is normally distributed with mean 76.343 and standard deviation 8.087; education follows a χ^2 distribution with 13.848 degrees of freedom; the intercept for the logistic model used to assign the probability of cognitive impairment (γ_0) is 0.707; and the standard deviation of the errors in the linear equation used to assign the value of the test score (σ) is 3.028.
